# Evaluation of Drain Insertion After Appendicectomy for Complicated Appendicitis: A Systematic Review and Meta-Analysis

**DOI:** 10.7759/cureus.32018

**Published:** 2022-11-29

**Authors:** Abduelraheim Abu, Ali Yasen Mohamedahmed, Amin Alamin, Mazin Mohamed, Mohamed Osman, Mohammed Jibreel Mohammed, Hiba Abdalla, Hazim A Eltyeb, Omer Ali, Rifat Mohamad, Safaa Hamid, Shaza Faycal Mirghani, Yousif Hamad, Hussam Khougali Mohamed

**Affiliations:** 1 General and Colorectal Surgery, Whipps Cross Hospital, London, GBR; 2 General Surgery, Royal Wolverhampton NHS (National Health Service) Trust, Birmingham, GBR; 3 General and Colorectal Surgery, London North West University Healthcare NHS (National Health Service) Trust, London, GBR; 4 General Surgery, University Hospitals Sussex NHS (National Health Service) Foundation Trust, Brighton, GBR; 5 General Surgery, Princess of Wales Hospital, Bridgend, GBR; 6 General and Colorectal Surgery, University Hospital Wishaw, Glasgow, GBR; 7 Vascular Surgery, Royal Stoke University Hospital, Stoke-on-Trent, GBR; 8 General Surgery, Health Education North East, Newcastle upon Tyne, GBR; 9 General and Colorectal Surgery, Queen Elizabeth The Queen Mother Hospital, Kent, GBR; 10 General Surgery, Wirral University Teaching Hospital NHS (National Health Service) Foundation Trust, Liverpool, GBR; 11 General and Colorectal Surgery, Kent and Canterbury Hospital, Kent, GBR; 12 General Surgery, Barnet Hospital, London, GBR; 13 General Surgery, Surrey and Sussex Healthcare NHS (National Health Service) Trust, Leeds, GBR; 14 General and Upper GI (Gastrointestinal) Surgery, University Hospital Hairmyres, Glasgow, GBR

**Keywords:** intra-abdominal collection, intra-abdominal abscess, postoperative ileus, drain insertion, ruptured appendicitis

## Abstract

This meta-analysis aims to evaluate the comparative outcomes of drain insertion versus no drain after appendicectomy for complicated appendicitis. A systematic search of PubMed, Cochrane Library and Scopus was conducted, and all studies comparing drain versus no drain after appendicectomy for complicated appendicitis were included. Abdominal collection, surgical site infection (SSI), bowel obstruction, faecal fistula, paralytic ileus, length of hospital stay (LOS) and mortality were the evaluated outcome parameters for the meta-analysis. Seventeen studies reporting a total number of 4,255 patients who underwent appendicectomy for complicated appendicitis with (n=1,580) or without (n=2,657) drain were included. There was no significant difference between the two groups regarding abdominal collection (odds ratio (OR)=1.41, P=0.13). No-drain group was superior to the drain group regarding SSI (OR=1.93, P=0.0001), faecal fistula (OR=4.76, P=0.03), intestinal obstruction (OR=2.40, P=0.04) and paralytic ileus (OR=2.07, P=0.01). There was a difference regarding mortality rate between the two groups (3.4% in the drain group vs 0.5% in the no-drain group, risk difference (RD)=0.01, 95% CI (-0.01, 0.04), P=0.36). In conclusion, this meta-analysis has shown that drains have no effect on the development of intra-abdominal collections in complicated appendicitis, but it can significantly increase the risk of postoperative complications such as fistula, surgical site infection (SSI), bowel obstruction, ileus and length of hospital stay.

## Introduction and background

Acute appendicitis is the commonest cause of surgical emergencies in the abdomen [1-3]. It carries a lifelong risk of 7%-8% [1,4,5]. It is classified as simple and complicated which is defined as the presence of an appendicular abscess, perforation or gangrene [6,7]. The diagnosis is made clinically, based on history, examination and laboratory findings. Where there is diagnostic uncertainty, radiological investigations such as ultrasound and CT scans are used [1,6]. It carries a higher risk of morbidity and mortality; for instance, the mortality rate following perforation is 5.1 per 1,000 [5,8].

Laparoscopic appendicectomy is considered the standard surgical management of acute appendicitis; this can be done either through a three-port or single-port approach [9-13]. Postoperative complications in complicated appendicitis include wound infection (20%), intra-abdominal abscess (9%-20%) and increased length of hospital stay [5,8,14-16]. Some surgeons prefer using prophylactic surgical drains routinely during appendicectomy for complicated appendicitis, while others avoid their use due to the controversy regarding their overall benefits and potential risks.

Surgical drains are used to reduce the rate of postoperative collections and abscess formation (1%-2% in simple appendicitis) [17]. However, they can cause considerable discomfort to the patients. Some evidence have suggested that surgical drains increase the risk of surgical site infection, among other problems such as erosion, obstruction, drain entrapment or loss due to displacement, kinking or migration [18]. Furthermore, in a meta-analysis done by Petrowsky et al., the authors have shown that surgical drains for complicated appendicectomies do not reduce the risk of postoperative complications, and they may increase the rate of other postoperative complications, including enterocutaneous fistula formation (4.2%-7.5%) and wound infection (43%-85%) [19]. Therefore, there is a debate on the overall benefits of using surgical drains routinely in appendicectomy. Our aim was to systematically review the literature and conduct a meta-analysis of the available relevant studies to assess the advantages of using drains versus no drains in complicated appendicitis.

## Review

This systematic review was designed, performed and reported as per the recommendations of the Cochrane Handbook for Systematic Reviews of Interventions and Preferred Reporting Items for Systematic Reviews and Meta-Analyses (PRISMA) guidelines for meta-analyses reporting [20,21]. Inclusion and exclusion criteria are shown in Table 1.

**Table 1:** Inclusion and exclusion criteria used in this meta-analysis.

Inclusion criteria	Exclusion criteria
Randomized controlled trials (RCTs) or comparative observational studies	Non-comparative studies, case series, case reports and letters
Including patients of all age groups of any gender	
Including patients with complicated acute appendicitis	
Including patients undergoing laparoscopic or open appendicectomy	
Including patients who received drain with appendicectomy	
Including patients who did not receive drain with appendicectomy	
Comparing the outcomes of the drain group with the no-drain group	

Search strategy

A literature search was performed by three authors independently in PubMed, Cochrane Library and Scopus up to June 2021. All studies of randomized controlled trials (RCTs) or comparative observational studies comparing the outcomes of drain or non-drain after open or laparoscopic appendicectomy for patients with complicated acute appendicitis were included in this analysis. Single-arm studies, case series and case reports were excluded. The keywords used in the search were “drain" [All Fields] AND (“drainage" [MeSH Terms] OR “drainage" [All Fields] OR “drain" [All Fields]) AND “laparoscopic appendicectomy" OR "laparoscopic appendectomy” [All Fields] OR “open appendicectomy” [All field] AND “acute appendicitis" [All Fields]. The searches were limited to human subjects, and no language or publication date restrictions were applied. The references of relevant reviews and included studies were also checked manually to identify additional potentially eligible studies.

Outcomes

Primary outcomes were the rates of postoperative abdominal collection formation. Postoperative complications including surgical site infection, bowel obstruction, faecal fistula, paralytic ileus, antibiotics duration in days, length of hospital stay (LOS) in days and mortality were considered secondary outcomes.

Study selection and data extraction

Three authors executed the literature, duplicated studies were excluded, the titles and abstracts were evaluated for relevance, and then records were classified as included, excluded or requiring further evaluation. All studies meeting the inclusion criteria of our study were included. Disagreements in the selection of studies were resolved by discussion between the reviewers. However, if the discrepancies remained unresolved, a fourth reviewer was involved.

All data were extracted manually and independently by three authors and revised by another three authors using Excel (2018, Microsoft Corporation, Washington, United States). The information collected from each study was author, year of publication, study design, number of populations in the study, presence of drain or no drain and comparative outcomes.

Assessment for risk of bias

**Figure 1:**
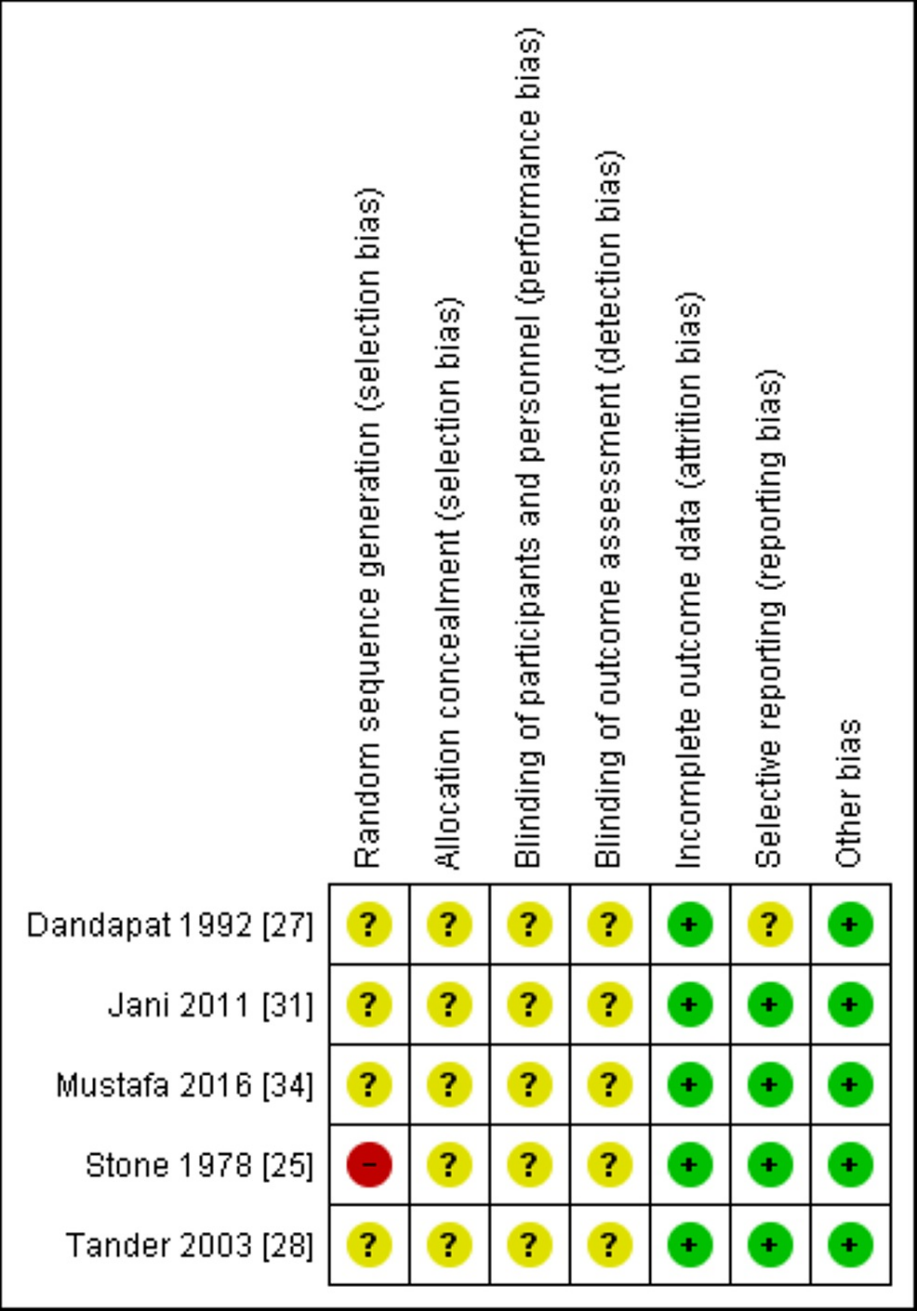
Risk of bias summary of included RCTs. RCT: randomized control trial.

**Figure 2:**
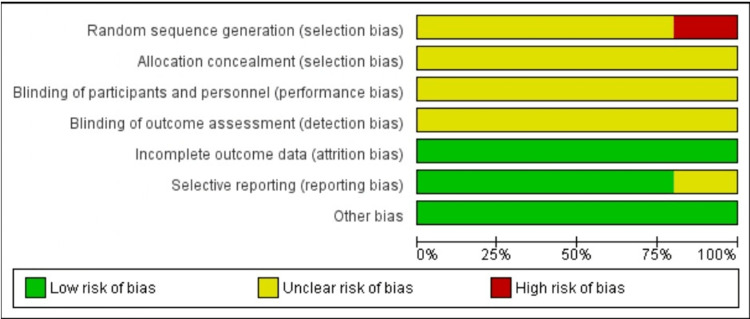
Risk of bias graph of included RCTs. RCT: randomized control trial.

The Cochrane risk of bias tool was used to appraise the risk of bias for the randomized trials [22]. Two investigators independently reviewed all studies and graded the risk as "high," "low" or "unclear" in the following categories: random sequence generation, allocation concealment, blinding of participants and personnel, blinding of outcome assessment, incomplete outcome data, selective reporting and other sources of bias. Newcastle-Ottawa scale (NOS) was used to assess the risk of bias in observational studies [23]. Studies were considered low, medium or high risk of bias if the NOS was 9, 7 or 8 or <6, respectively. Disagreement was resolved with discussion and consultation of a third reviewer (Figures 1, 2).

Statistical analysis

Meta-analysis was performed using Review Manager (RevMan) Version 5.4, The Cochrane Collaboration, 2020. Dichotomous outcomes were pooled with a random-effects model to estimate the odds ratio (OR) or risk difference (RD) (where more than three studies reported zero events in both drain and no-drain groups) with 95% confidence interval. For continuous outcomes, a mean difference (MD) with 95% CI was estimated. When mean values were not available for continuous outcomes, data on median and interquartile range (IQR) were extracted and subsequently converted to mean and standard deviation (SD) using the well-practised equation described by Hozo et al. [24].

The results were considered statistically significant if the P-value was <0.05 or if the 95% CI did not include 1.00. Heterogeneity evaluated using the Cochran Q test (χ^2^) was used to evaluate the heterogeneity and I^2^ was reported to quantify it: a value of 0 % indicated no heterogeneity and over 50% indicated significant heterogeneity. Subgroup analysis for studies that included only laparoscopic appendicectomy was considered if the outcome was reported in three or more studies. To check possible causes of heterogeneity and evaluate the robustness of the results, sensitivity analysis was performed by calculating the risk ratio or RD for dichotomous variables. Moreover, the analysis was repeated leaving one study out each time to check the effect of each study on heterogeneity. 

Results

**Figure 3:**
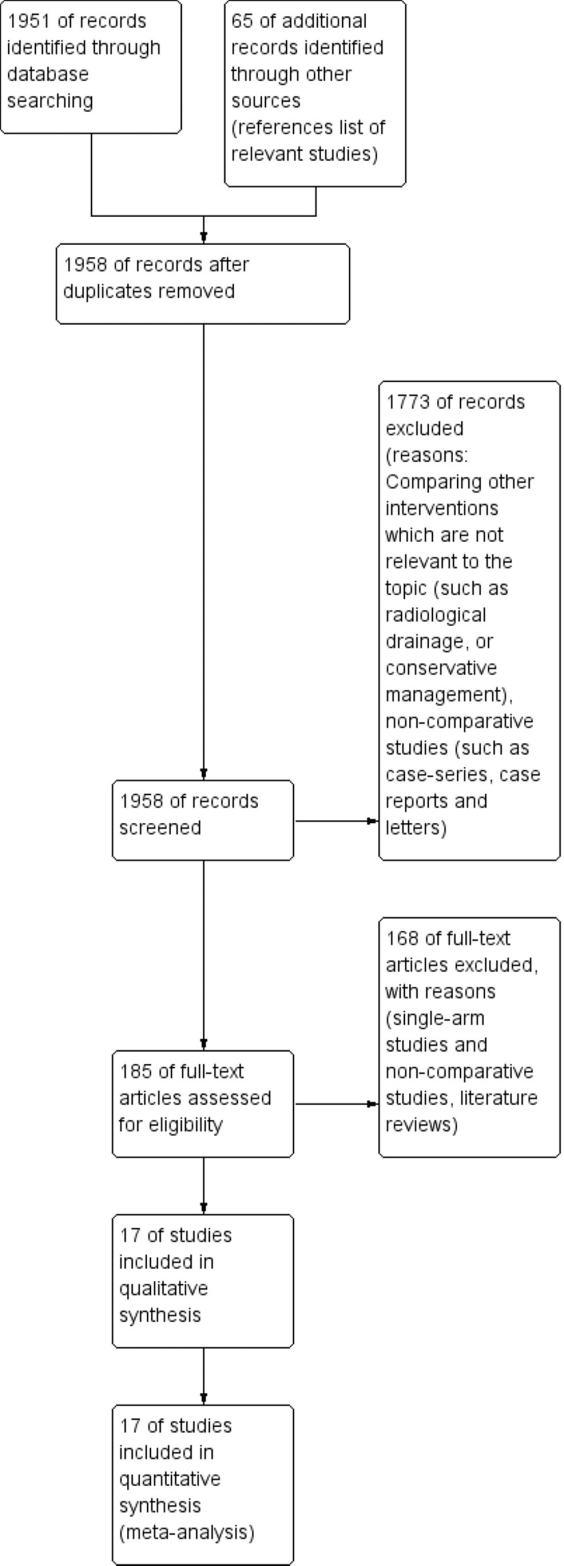
Preferred Reporting Items for Systematic Reviews and Meta-Analyses (PRISMA) flow chart.

A total number of 1,958 studies were identified after the systemic search of the above-mentioned databases. After reviewing titles/abstracts and excluding duplicates, 1,773 studies were excluded leaving 185 studies. Full manuscripts were reviewed and assessed for eligibility criteria resulting in 17 studies that were included in the analysis [25-41]. The Preferred Reporting Items for Systematic Reviews and Meta-Analyses (PRISMA) flow chart is shown in Figure 3. These 17 studies included 4,237 patients, 1,580 had had a drain inserted intraoperatively and 2,657 did not have a drain. Included studies' characteristics are shown in Table 2.

**Table 2:** Characteristics of included studies. DG: drain group, NDG: no-drain group, RCT: randomized controlled trial, NA: not available.

Study	Country	Type of the study	Number of patients	Type of operation	Inclusion/exclusion criteria and definition of complicated appendicitis
Stone et al., 1978 [25]	Georgia	Retrospective cohort	Total: 94; DG: 49; NDG: 45	Open	Inclusion and exclusion criteria: NA. Definition of complicated appendicitis: gangrenous or perforated appendicitis
Greenall et al., 1978 [26]	United Kingdom	RCT	Total: 103; DG: 48; NDG: 55	Open	Exclusion criteria: appendicular mass/abscess. Definition of complicated appendicitis: gangrenous appendicitis, associated with turbid infected fluid
Dandapat and Panda, 1992 [27]	India	RCT	Total: 86; DG: 40; NDG: 46	NA	NA
Tander et al., 2003 [28]	Turkey	RCT	Total: 140; DG: 70; NDG: 70	Open	Inclusion criteria: paediatric cases with uncomplicated perforated appendicitis. Exclusion criteria: appendicular mass/abscess. Definition of complicated appendicitis: gross or microscopic evidence of appendicular perforation with no more discolouration of peritoneal fluid after washing out
Narci et al., 2007 [29]	Turkey	Retrospective cohort	Total: 226; DG: 109; NDG: 117	Open	Inclusion criteria: children with macroscopic perforation. Exclusion criteria: appendix could not be visualized and drained without appendectomy. Definition of complicated appendicitis: macroscopic perforation
Allemann et al., 2011 [30]	Switzerland	Case match study	Total: 260; DG: 130; NDG: 130	Laparoscopic	Exclusion criteria: simple acute appendicitis (i.e., no peritoneal reaction), generalized peritonitis, preoperatively known immunodeficiencies, aged <16 years and incomplete dataset. Definition of complicated appendicitis: localized peritonitis, perforation of the appendix, presence of pus or fibrin membranes around the appendix or frank peri-appendicular abscess
Jani and Nyaga, 2011 [31]	Kenya	RCT	Total: 90; DG: 45; NDG: 45	Open	Inclusion criteria: advanced appendicular pathology and aged over 13 years. Exclusion criteria: simple acute appendicitis or laparoscopic appendicectomy. Definition of complicated appendicitis: perforated, mass or phlegmon
Pakula et al., 2014 [32]	USA	Retrospective cohort	Total: 148; DG: 43; NDG: 105	Laparoscopic	Inclusion criteria: patients with the diagnosis of gangrenous or perforated appendicitis based on the review of pathology and operative reports. Exclusion criteria: simple or suppurative appendicitis and those treated with interval appendectomy. Definition of complicated appendicitis: gangrenous and perforated appendicitis
Song and Jung, 2015 [33]	Korea	Retrospective cohort	Total: 342; DG: 108; NDG: 234	Open: 181; Laparoscopic: 161	Inclusion criteria: children under 18 years old who were diagnosed with acute appendicitis. Definition of complicated appendicitis: perforated appendicitis
Schlottmann et al., 2016 [35]	Argentina	Retrospective cohort	Total: 225; DG: 169; NDG: 56	Laparoscopic	Inclusion and exclusion criteria: NA. Definition of complicated appendicitis: intraoperatively as the presence of gangrenous/perforated appendicitis with peritonitis
Abdulhamid and Sarker, 2018 [36]	Iraq	Retrospective cohort	Total: 227; DG: 114; NDG: 113	Open	Inclusion criteria: open appendectomy for complicated appendicitis irrespective of age. Definition of complicated appendicitis: perforated with localized abscess formation
Aneiros Castro et al., 2018 [37]	Spain	Retrospective cohort	Total: 192; DG: 79; NDG: 63	Laparoscopic	Inclusion criteria: patients with perforated appendicitis. Exclusion criteria: incidental appendectomy during another laparoscopic surgical procedure and those treated with interval appendectomy. Definition of complicated appendicitis: identifiable macroscopic hole in the appendix during the surgery
Miranda-Rosales et al., 2019 [38]	Peru	Retrospective cohort	Total: 150; DG: 50; NDG: 100	Open	Inclusion criteria: aged >18 years with complicated appendicitis. Exclusion criteria: laparoscopic appendicectomy, patients on anticoagulation, immunocompromised and pregnancy. Definition of complicated appendicitis: localized or generalized peritonitis and appendicular abscess
Fujishiro et al., 2021 [39]	Japan	Propensity-matched study	Total: 1,762; DG: 485; NDG: 1304	Open: 346; Laparoscopic: 958	Inclusion criteria: complicated appendicitis in children (aged 15 years and below). Exclusion criteria: interval appendectomies. Definition of complicated appendicitis: perforation, gangrene or intra-abdominal abscess
Nazarian et al., 2021 [40]	UK	Retrospective cohort	Total: 76; DG: 26; NDG: 50	Laparoscopic	Inclusion criteria: over the age of 16 with complicated appendicitis. Exclusion criteria: caecal/appendicular malignancy. Definition of complicated appendicitis: histologically proven gangrenous or perforated appendicitis
Schmidt et al., 2020 [41]	Germany	Retrospective cohort	Total: 65; DG: 32; NDG: 33	Open: 11; Laparoscopic: 55	Inclusion criteria: age range from two to 17 years who presented with perforated appendicitis. Exclusion criteria: severe neurological dysfunction and inflammatory bowel disease. Definition of complicated appendicitis: perforated appendicitis on histology
Mustafa et al., 2020 [34]	Pakistan	RCT	Total: 68; DG: 34; NDG: 34	Open	Exclusion criteria: immunocompromised patients and those with generalized peritonitis (perforated appendix with pus in three or more quadrants of the abdominal cavity visible per-operatively). Definition of complicated appendicitis: perforated appendicitis intra-operatively

Regarding age group, seven studies had only paediatrics [28-30,33,37,39,41]. Three had only adults [31,38,40], and the rest had a mixture of both adults and paediatrics population [25-27,32,34-36]. The baseline characteristics were found comparable (Table 3).

**Table 3:** Baseline characteristics of population. DG: drain group, NDG: no-drain group, WBCS: white blood cell count, NA: not available, SD: standard deviation, IQR: interquartile range.

Study	Age (years): Mean±SD/Median (range/IQR)	Male:female ratio	Drain removal (days) Mean±SD	WBCS count: Mean±SD/Median (range/IQR)	Antibiotics duration (days): Mean±SD/Median (range/IQR)
Stone et al., 1978 [25]	All: 23.2 (0.3-82)	All: 159:124	5-10	NA	NA
Greenall et al., 1978 [26]	NA	DG: 31:17; NDG: 27:28	NA	NA	NA
Dandapat and Panda, 1992 [27]	NA	NA	NA	NA	NA
Tander et al., 2003 [28]	DG: 6.89±3.5; NDG: 7.31±3.4	DG: 50:20; NDG: 52:18	1	NA	DG: 5; NDG: 5
Narci et al., 2007 [29]	DG: 8.7±3.3; NDG: 8.5±3.6	DG: 75:34; NDG: 75:42	NA	DG: 18.4±5.8; NDG: 18.4±7.0	DG: 9.5±5.5; NDG: 7.7±2.7
Allemann et al., 2011 [30]	DG: 38 (16-75); NDG: 31 (16-71)	DG: 72:58; NDG: 83:67	2	DG: 14.0 (4-28.3); NDG: 14.3 (4.1-23.6)	
Jani and Nyaga, 2011 [31]	13-26 years; DG: 47.73%; NDG: 52.27%; 27-52 years; DG: 52.17%; NDG: 47.83%	DG: 25:20; NDG: 21:24	3	NA	DG: 5; NDG: 5
Pakula et al., 2014 [32]	DG: 32±14; NDG: 29±10	DG: 33:10; NDG: 25:80	9±5.4	DG: 15±4.8; NDG: 15.5±5	DG: 6.2±4; NDG: 6.6±4
Song and Jung, 2015 [33]	DG: 9.92±4.25; NDG: 10.97±4.04	DG: 55.6%:44.4%; NDG: 60.3%:39.7%	NA	DG: 16.7±5.96; NDG: 15.7±4.57	DG: 6.38±3.6; NDG: 3.87±2.38
Schlottmann et al., 2016 [35]	DG: 43.3 (16-92); NDG: 43.1 (16-93)	DG: 36:20; NDG: 98:71	NA	DG: 14.4 (6.3-23.4); NDG: 15.3 (4.4-36.1)	NA
Abdulhamid and Sarker, 2018 [36]	DG: 31.75; NDG: 30.77	DG: 47%-53%; NDG: 53%:47%	NA	NA	NA
Aneiros Castro et al., 2018 [37]	DG: 7.57±3.5; NDG: 8.07±3.2	All: 63.1%:36.9%	NA	NA	DG: 7.51; NDG: 6.61
Miranda-Rosales et al., 2019 [38]	DG: 35 (15-72); NDG: 36.76 (15-70)	DG: 60:40; NDG: 60:40	NA	DG: 15.3 (10.0-19.0); NDG: 15.6 (10.9-19.0)	NA
Fujishiro et al., 2021 [39]	NA	DG: 55.9:44.1; NDG: 59.9:40.1	NA	NA	NA
Nazarian et al., 2021 [40]	DG: 39.62 (17-82); NDG: 37.42 (19-79)	NA	NA	DG: 15.1; NDG: 14.6	NA
Schmidt et al. 2020 [41]	DG: 10.34; NDG: 10.70	DG: 18:14; NDG: 16:17	NA	DG: 16.68; NDG: 16.62	
Mustafa et al., 2020 [34]	DG: 26.41±6.24; NDG: 26.74±4.97	DG: 58.8%:41.1%; NDG: 47.1%:52.9%	NA	NA	DG: 5; NDG: 5

Methodological appraisal of included studies

The methodological appraisal of the included observational studies [26,29,30,32,33,34-41] is illustrated in Table 4. The risk of bias was judged as low in two studies [36,39] and moderate in the rest of the observational studies. An overview of the risk of bias of the included RCTs [25,27,28,31,34] is shown in Figures 1, 2. None of the included RCTs reported random sequence generation and allocation concealment resulting in an unclear or high risk of selection bias. Moreover, there was an unclear risk of performance and detection bias due to a lack of blinding. Attrition bias and reporting bias were judged to be unclear in one study [27]; however, the rest of included randomized control trials (RCTs) showed a low risk of bias in these domains.

**Table 4:** Methodological quality of the observational studies assessed with the Newcastle-Ottawa scale. Symbol (*) is the number of points given to each study according to the Newcastle-Ottawa scale (* gives one point, and ** gives two points).

Study	Is the case definition adequate?	Representativeness of the cases	Selection of controls	Definition of controls	Comparability of cases - controls on the basis of the design or analysis	Ascertainment of exposure	Same method of ascertainment for cases - controls	Non-response rate	Total
Stone et al., 1978 [25]	*	*		*	*	*	*	*	7
Narci et al., 2007 [29]	*	*		*	**	*	*	*	8
Allemann et al., 2011 [30]	*	*		*	**	*	*	*	8
Pakula et al., 2014 [32]	*	*		*	**	*	*	*	8
Song and Jung, 2015 [33]	*	*		*	*	*	*	*	7
Schlottmann et al., 2016 [35]	*	*		*	**	*	*	*	8
Abdulhamid and Sarker, 2018 [36]	*	*	*	*	**	*	*	*	9
Aneiros Castro et al., 2018 [37]	*	*		*	**	*	*	*	8
Miranda-Rosales et al., 2019 [38]	*	*		*	**	*	*	*	8
Fujishiro et al., 2021 [39]	*	*	*	*	**	*	*	*	9
Nazarian et al., 2021 [40]	*	*		*	**	*	*	*	8
Schmidt et al., 2020 [41]	*	*		*	**	*	*	*	8

Primary outcome

Abdominal Collection

**Figure 4:**
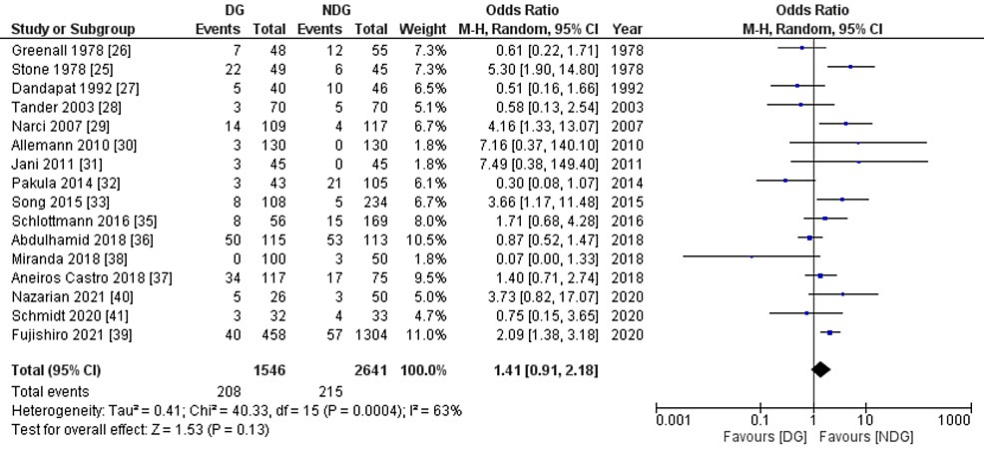
Forest plot of abdominal collection. DG: drain group, NDG: no-drain group, M-H: Mantel-Haenszel.

The postoperative abdominal collection rate was reported in 16 studies with a total number of 4,007 patients; 10.5% of them developed postoperative abdominal collection (Figure 4). The difference was not statistically significant between the drain and no-drain group (13.4% vs 8.1%, OR=1.41, 95% CI (0.91,2.18), P=0.13). The level of heterogeneity between included studies was high (I^2^=63%, P=0.0004).

Subgroup for Open and Laparoscopic Appendicectomy

**Figure 5:**
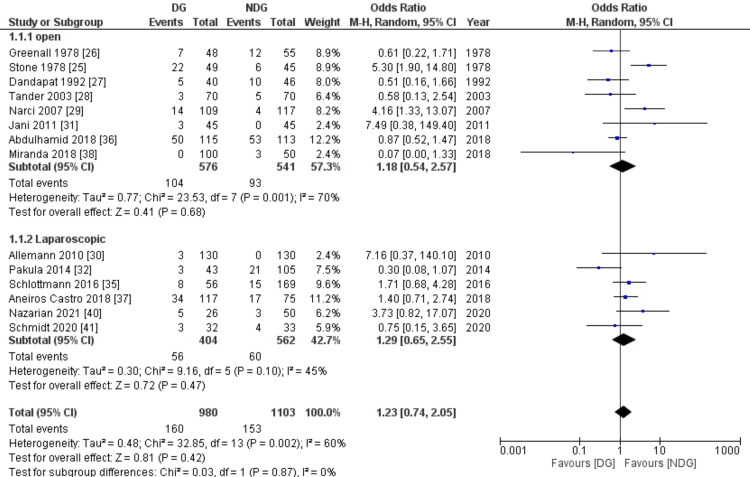
Forest plot of subgroup analysis for open and laparoscopic appendicectomy. DG: drain group, NDG: no-drain group, M-H: Mantel-Haenszel.

The difference in abdominal collection rate remains non-significant between the drain and no-drain groups when subgroup analysis for open and laparoscopic appendicectomy was applied ((OR=1.18, 95% CI (0.54, 2.57), P=0.68) and (OR=1.29, 95% CI (0.65, 2.55), P=0.47), respectively) (Figure 5).

Subgroup for Paediatrics-Only Studies

**Figure 6:**
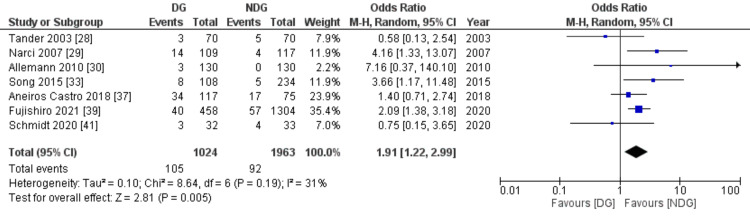
Forest plot of the subgroup for paediatrics-only studies. DG: drain group, NDG: no-drain group, M-H: Mantel-Haenszel.

Analysis of the studies that include only paediatrics patients, seven studies with 2,987 children, revealed that the rate of postoperative collection is statistically lower in the no-drain group compared to the drain group (4.7% vs 10.2%, OR=1.91, 95% CI (1.22, 2.99), P=0.005) (Figure 6).

Postoperative Complications

**Figure 7:**
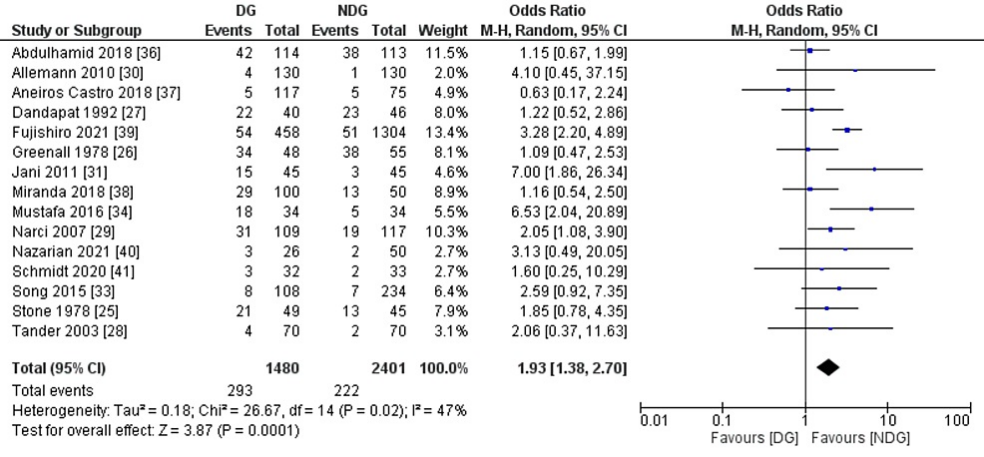
Forest plot of surgical site infection. DG: drain group, NDG: no-drain group, M-H: Mantel-Haenszel.

**Figure 8:**
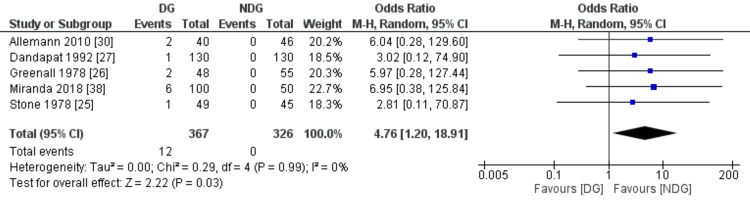
Forest plot of faecal fistula. DG: drain group, NDG: no-drain group, M-H: Mantel-Haenszel.

**Figure 9:**
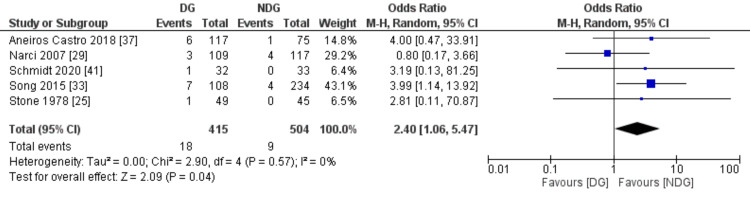
Forest plot of bowel obstruction. DG: drain group, NDG: no-drain group, M-H: Mantel-Haenszel.

**Figure 10:**
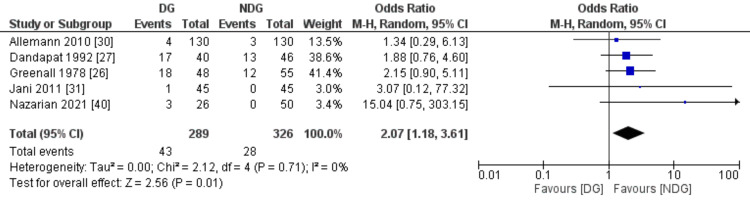
Forest plot of paralytic ileus. DG: drain group, NDG: no-drain group, M-H: Mantel-Haenszel.

The measured postoperative complication outcomes were SSI, faecal fistula, bowel obstruction and paralytic ileus (Figures 7-10). All these outcomes were significantly higher in the drain group when compared to the no-drain group. SSI rate was 19.8% in the drain group and 9.2% in the no-drain group (OR=1.93, 95% CI (1.38,2.70), P=0.0001). Regarding faecal fistula, it was reported in 3.2% of patients in the drain group, while no patient developed fistula in the no-drain group (OR=4.76, 95% CI (1.20, 18.91), P=0.03). Intestinal obstruction was reported in five studies with the rate being statistically higher in the drain group when compared to the no-drain group (4.3% vs 1.8%, OR=2.40, 95% CI (1.06, 5.47), P=0.04). Paralytic ileus rate was 14.9% and 8.6% in the drain and the no-drain groups, respectively (OR=2.07, 95% CI (1.18, 3.61), P=0.01).

Subgroup for Laparoscopic Appendicectomy

Subgroup analysis for studies that include only laparoscopic appendicectomy cases for SSI was consistent with all studies analysis (OR=2.18, 95% CI (1.05, 4.52), P=0.04).

Length of Hospital Stay

**Figure 11:**
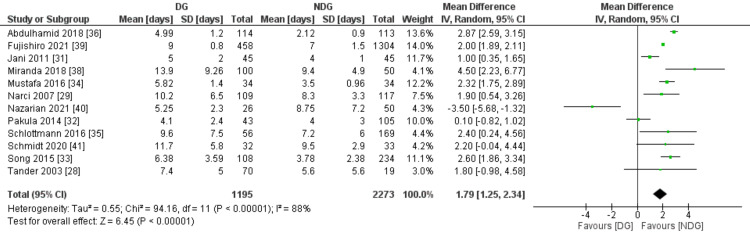
Forest plot of length of hospital stay. DG: drain group, NDG: no-drain group, M-H: Mantel-Haenszel.

Length of hospital stay was mentioned in 12 studies including 3,468 patients (Figure 11). The no-drain group showed a statistically significant shorter length of hospital stay (LOS) when compared to the drain group (MD=1.79, 95% CI (1.25, 2.34), P=0.00001). A high level of heterogeneity was observed between included studies (I^2^=88%, P=0.00001).

Mortality

**Figure 12:**
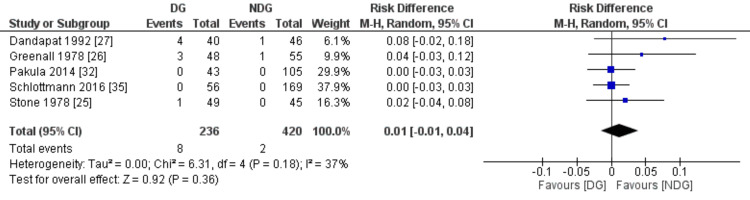
Forest plot of mortality. DG: drain group, NDG: no-drain group, M-H: Mantel-Haenszel.

Mortality was reported in five studies with a total number of 656 patients (Figure 12). There was a difference regarding mortality rate between the two groups (3.4% in the drain group vs 0.5% in the no-drain group, RD=0.01, 95% CI (-0.01, 0.04), P=0.36). The level of heterogeneity was low between the included studies (I^2^=37%, P=0.18).

Sensitivity analysis

There was no change in the direction of the pooled effect size when RD or RR was applied for dichotomous outcomes. Similarly, excluding one study at a time analysis has not shown significant discrepancies with the original analysis.

Discussion

Acute appendicitis is a very common condition with a lifetime risk of 7% [2]. Although appendicectomy is recognized as one of the commonest surgical procedures, there is around a 9% chance of having a complication related to appendicectomy and mortality ranges between 0.24% and 4% [7,16-17]. SSIs including wound infection and postoperative collection are the commonest to happen after complicated appendicitis compared to simple appendicitis [8,14,15]. However, there is another wide range of postoperative complications related to complicated appendicitis such as ileus, obstruction, stumpitis, dehiscence, fistula formation and rarely death [42].

Acute uncomplicated appendicitis is a term usually used to describe a localized inflammation of the appendix with no localized or generalized effect or extension to adjacent structures [10-16]. As a rule of thumb, complicated appendicitis includes perforated appendicitis which can include localized contamination, abscess formation and single or more quadrant contamination with pus or faeces; it also includes appendicular mass, fistulation and intestinal obstruction [43]. In 2012, a proposed intraoperative classification system was proposed by Gomes et al. [44]. This classification ranged from grade 0 being a normal looking appendix to grade 5 being diffuse peritonitis with a range of the severity of the inflammation in-between. Despite that, this classification has not been adopted widely.

Our meta-analysis has found no significant difference in the postoperative intra-abdominal collection between the two groups which is about the same in most of the included studies [35-37,39]. However, Song and Jung and Pakula et al. have found a statistical difference in high-rate intra-abdominal collection and abscess formation in their drain group patients [32,33]. This variation in the rate of intra-abdominal/pelvic collection postoperatively may attribute to many reasons such as too small a drain diameter to drain thick fluids, infection introduced by drain materials or blockage of the drain by a clot or debris [38].

The rate of the intra-peritoneal collection after complicated appendicitis was reported to be 8.6% in the literature; hence, the use of a surgical drain following surgery has shown to be of no significant benefit for complicated appendicitis, which is consistent with our meta-analysis finding (10.1%) [45]. Otherwise, in other surgeries where a high risk of the intra-abdominal collection can result in a high morbidity and mortality rate, a surgical drain should be used.

The commonly reported risk factors for collection are perforated appendicitis, high total leucocyte count and duration of symptoms [46,47]. Irrigation of the peritoneal cavity was found to be an increasing risk of development of the intra-abdominal collection, and the pus can spread during washout from the localized area of infection in the right iliac fossa to the other compartments in the abdomen resulting in generalized peritonitis [47]. Moreover, a recent systematic review concluded that suction only is not inferior to suction and irrigation in terms of the development of the postoperative collection and the adoption of a suction-only approach may reduce the operative time [45,48]. Assessment of the effect of these known risk factors for the collection was not possible in this review due to the lack of data in the included studies.

Interestingly, the incidence of the post-appendicectomy collection was found to be higher in laparoscopic appendicectomy when compared to the open approach in the Cochrane meta-analysis that was performed in 2002; however, in the subsequent update of the same review in 2004 and 2010, the likelihood of intra-abdominal collection decreased from OR=2.77 in 2002 to OR=1.87 in 2010 [48,49].

Several reports suggested that drain can be one of the causes of intestinal obstruction and paralytic ileus during the postoperative period, which eventually can result in a delayed postoperative recovery [50]. This can be either mechanical obstruction due to the presence of the drain as a foreign body inside the abdominal cavity or due to infection and subsequent scarring that can be introduced through the drain from outside the abdomen [51]. This review showed statistically significant differences in the rate of postoperative bowel obstruction, ileus and fistula formation in the drain group compared to the no-drain group. This is significantly related to the high morbidity rate and delayed recovery noted in the drain group. The development of postoperative enteric fistula after appendicectomy represents only 0.5% of cases; this can be due to either a leak from appendicular stump, iatrogenic injury to the caecum or presence of distal bowel obstruction [52,53]. As per the results that previously mentioned and analysed in this review, the absence of a drain was of significant benefit for the length of hospital stay (LOS) than for the use of a drain. The hospitalization is significantly shorter in the no-drain group which has a positive effect on patient quality of care and the cost of the health system in general. In addition to that, early discharge prevents patients from hospital-acquired infections and promotes psychological well-being [54]. We can argue that patients with complicated appendicitis and drain insertion generally would have a higher risk of developing postoperative complication and prolonged hospital stay.

Prophylactic drain after abdominal operations has been studied extensively in the literature. A Cochrane review concluded that it is unclear whether routine abdominal drainage had any effect on the reduction of mortality at 30 days or postoperative complications after pancreatic surgery [55]. Regarding colorectal surgery, the recommendation of a recent meta-analysis of randomized controlled trials was against the usage of the drain as there was no difference in anastomotic leak rate, morbidity and mortality between the drain and no-drain groups [56]. Moreover, the recommendation remains against the drain after laparoscopic cholecystectomy and liver resection [57,58].

The present meta-analysis is the most comprehensive meta-analysis of the literature to date. In 2015, a Cochrane review of five studies compared drain versus no drain in complicated appendicitis [59]. The authors reported that there is no difference between the two groups regarding the intra-abdominal collection, SSI and mortality, which is in line with our results. However, the authors have not compared the outcomes regarding bowel obstruction, fistula, ileus and antibiotics duration. This should be taken into account that our pooled population size was much larger than the aforementioned meta-analysis, indicating that our findings may be less subject to type 2 error.

Limitations 

There are obvious limitations in included studies as most of them are observational studies and not well-designed RCTs which are inevitably subject to selection bias. Due to the lack of available data, we were not able to conduct subgroup analysis for the types of complicated appendicitis (perforated appendicitis, appendicular mass, etc) or the immunocompromised and age extremities patients (paediatrics vs elderly) as these factors might have biased our findings in favour of an intervention. Moreover, the reported outcomes by the included studies, except for bowel obstruction, fistula and ileus, have been heterogeneous. Authors recommend to shed more light on the lack of a uniform definition or proper scoring system for complicated appendicitis in the literature; hence, an agreement about setting a clear definition and scoring system for complicated appendicitis should be adopted for the future research and trials in order to meet the demand of the fast-growing medical care.

## Conclusions

This meta-analysis has shown that drains do not affect the development of intra-abdominal collections in complicated appendicitis, but they can significantly increase the risk of postoperative complications such as fistula, surgical site infection (SSI), bowel obstruction, ileus and length of hospital stay (LOS). 
